# Exosomes Engineering and Their Roles as Therapy Delivery Tools, Therapeutic Targets, and Biomarkers

**DOI:** 10.3390/ijms22179543

**Published:** 2021-09-02

**Authors:** Nika Kučuk, Mateja Primožič, Željko Knez, Maja Leitgeb

**Affiliations:** 1 Faculty of Chemistry and Chemical Engineering, University of Maribor, Smetanova 17, 2000 Maribor, Slovenia; nika.kucuk@um.si (N.K.); mateja.primozic@um.si (M.P.); zeljko.knez@um.si (Ž.K.); 2 Faculty of Medicine, University of Maribor, Taborska Ulica 8, 2000 Maribor, Slovenia

**Keywords:** exosomes, therapeutic biomaterials, cargo incorporation, therapeutic applications, drug delivery

## Abstract

Exosomes are becoming increasingly important therapeutic biomaterials for use in a variety of therapeutic applications due to their unique characteristics, especially due to the ineffectiveness and cytotoxicity of some existing therapies and synthetic therapeutic nanocarriers. They are highly promising as carriers of drugs, genes, and other therapeutic agents that can be incorporated into their interior or onto their surface through various modification techniques to improve their targeting abilities. In addition, they are biocompatible, safe, and stable. The review focuses on different types of exosomes and methods of their preparation, including the incorporation of different kinds of cargo, especially for drug delivery purposes. In particular, their importance and effectiveness as delivery vehicles of various therapeutic agents for a variety of therapeutic applications, including different diseases and disorders such as cancer treatment, cardiovascular and neurodegenerative diseases, are emphasized. Administration routes of exosomes into the body are also included. A novelty in the article is the emphasis on global companies that are already successfully developing and testing such therapeutic biomaterials, with a focus on the most influential ones. Moreover, a comparison of the advantages and disadvantages of the various methods of exosome production is summarized for the first time.

## 1. Introduction

Various diseases and disorders, as well as conventional drug dosage forms, have led to the development of improved treatment pathways, one of which is drug delivery using a variety of nanocarriers [1,2]. Drug delivery is defined as the introduction of therapeutic drugs or active compounds into the human body through various administration routes, thereby achieving the desired therapeutic effect, improving efficiency and safety, as well as controlling the time and amount of drug release at the target location [2,3]. Such methods have the ability to improve the chemical stability of active compounds and improve their solubility. A smaller amount of drugs can be used and thus reduce any possible side effects and toxicity that may be present when using conventional therapies [3,4]. Conventional drug administration has some drawbacks, such as repeated drug dosing, where it is also difficult to achieve a specific target with a predicted concentration, uncontrolled drug release, and low bioavailability. Therefore, conventional drug administration has been used less and less recently, and a growing number of approaches have been developed to form different nanoparticles as nanocarriers suitable for drug delivery due to the programmed release of drugs to specific target locations [1,4].

Drug nanocarriers must ensure maximum efficacy, and therefore they need to be properly constructed and must have certain properties, such as biodegradability, non-immunogenicity, stability, ease of design, and delivery of cargo only to specific target cells or tissues [5]. They typically do not exceed a size of 100 nm and are non-toxic as they are made from biocompatible components. They have the ability to be selective and deliver the drug appropriately to diseased cells and not to healthy ones. Examples of these nanocarriers are liposomes, niosomes, micelles, dendrimers, and nanofibers [6], as well as exosomes [7].

Exosomes have become highly important nanocarriers due to their biocompatibility and safety [8]. In the past, scientists were convinced that exosomes were only cell debris without any important function, and their significance has only been discovered in the last two decades [9]. Exosomes are highly biostable, even in long-distance cell-to-cell communication [7]. They are able to deliver their cargo to recipient cells owing to the fact that the surface of exosomes has a specific molecular composition [10]. Additionally, they have the ability to stay unaltered through the digestive system, including when exposed to different digestive enzymes and other body fluids [8]. Therefore, exosomes are suitable carriers for the delivery of different biologically active compounds and other components that are easily degraded, as they can be incorporated into exosomes [11,12]. 

Exosomes are considered to be nanoparticles due to their size, ranging from 30–100 nm, and some of their properties, which are similar to nanoparticles, such as passive targeting and increased permeability and retention effects [13]. There are numerous types of cells that secrete these nanovesicles. Their important function involves intercellular communication by transferring different molecules, such as proteins, lipids, nucleic acids (DNAs and RNAs), and metabolites. The cargo of exosomes produced from different cells varies greatly [7,14,15]. Exosomes are produced from multivesicular bodies, which are considered to be formed by the budding of the plasma membrane, and when fusion between multivesicular bodies and the plasma membrane occurs, exosomes are secreted from the cells into the extracellular environment [7,15,16,17]. Exosomes serve as delivery vehicles of incorporated drugs and other active compounds, which can act synergistically with naturally occurring components in exosomes [18].

Not all the components present in naturally isolated exosomes are necessary for specific drug delivery, and therefore the use of synthetic therapeutic biomaterials, such as modified and synthetic exosomes, may be better for efficient drug delivery, as they are pure and precisely characterized nanocarriers [18,19].

Modified naturally isolated exosomes and synthetic exosomes that are completely artificial and produced in the laboratory are considered to be synthetic therapeutic biomaterials. Modified exosomes can be altered before their isolation with pre-isolation modifications or after their isolation with post-isolation modifications. Synthetic exosomes can also be produced through two different methodologies. They can be produced through a cell-based approach from larger substrates, which are then reduced to smaller units, and thus vesicles can be formed, or through a lipid membrane bilayer formation approach where individual molecules are used as substrates that assemble into complex structures [18,20].

Exosomes can be used for a variety of purposes, namely as cargo delivery vehicles, biomarkers, and therapeutic agents for the identification of various diseases, and in the development of new vaccines, mainly for cancer treatment [21,22,23]. 

This review article presents the growing importance of therapeutic biomaterials—exosomes in a variety of biomedical applications, primarily for use as delivery vehicles for drugs and other therapeutic agents. The division of exosomes and their isolation, modification, and production techniques are briefly explained. The article also includes a short schematic overview of the incorporation of cargo into exosomes, divided by hydrophobicity or hydrophilicity, which has not been presented in detail so far. In contrast, in other review articles the division was made mainly according to the exosomes’ source or incorporation method. For the first time, the preparation of exosomes on an industrial scale, where commercial companies that have already successfully established various exosome platforms and are already producing exosomes for therapeutic purposes, is presented. The most influential companies and their exosome products are also described, with an emphasis on their intended use. Special attention is also given to possible administration routes of therapeutic biomaterials. A comparison of the advantages and disadvantages of the various methods of exosome production and preparation are also given for the first time.

## 2. Exosomes

**Figure 1:**
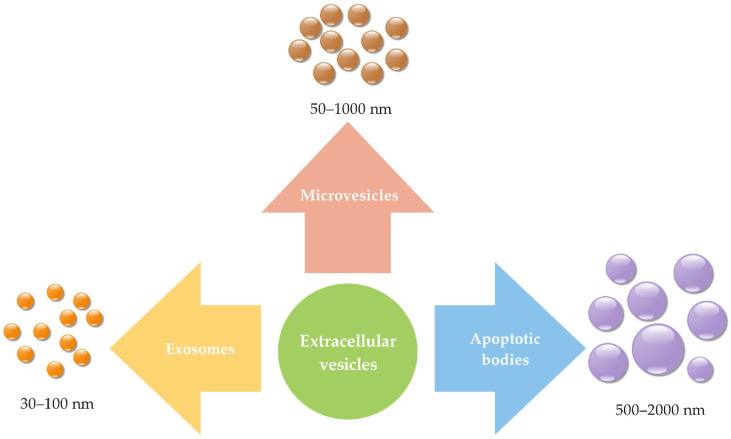
Classification of extracellular vesicles according to their size into exosomes (size diameter around 30–100 nm), microvesicles (size diameter around 50–1000 nm), and apoptotic bodies (size diameter around 500–2000 nm; summarized from [14,16,25,26]).

Exosomes are classified as extracellular vesicles (EVs), among which they are the smallest. EVs also include microvesicles (50–1000 nm) and apoptotic bodies (500–2000 nm) [14], as shown in Figure 1. They are classified according to their size, intracellular origin [24], and biophysiological properties [25]. 

Microvesicles, also known as ectosomes [27], are formed through the process of membrane budding, followed by fission of the vesicle from the surface of the cell [25]. Therefore, microvesicles contain proteins present in the plasma membrane and also cytosolic proteins, nucleic acids, and different metabolites [27].

Vesicles released from dying cells by a process called disassembly of apoptotic cells during apoptosis (programmed cell death) are apoptotic bodies. Apoptotic bodies are the largest extracellular vesicles and contain the remains of dying cells, including the plasma membrane, as well as nuclear and cytoplasmic material. They can also transmit their content through cell-to-cell communication [25,26,28,29].

As mentioned before, exosomes are the smallest among extracellular vesicles. Zhang et al. [30] classified exosomes based on asymmetric flow field-flow fractionation technology. They discovered two subpopulations of exosomes and named them large exosomes (90–120 nm) and small exosomes (60–80 nm), as well as specific non-membranous nanoparticles named exomeres (~35 nm).

Cells can secrete specific subpopulations of exosomes with a certain size and composition of proteins and nucleic acids, which strongly affects recipient cells [31].

The composition of exosomes coincides with the composition of the cell from which they are secreted; they also have the same regulated sorting mechanism [32]. The interior and surface of exosomes contain various bioactive compounds, including proteins, enzymes, receptors, growth factors, transcription factors, nucleic acids (mRNA, miRNA, DNA), lipids, and other metabolites [32,33,34]. The lipid composition includes cholesterol, phosphatidylserine, sphingomyelin, and saturated fatty acids. Among proteins, they contain the cytoplasmic, plasma, intracellular proteins, and nucleoprotein [35]. On the other hand, the membrane of exosomes normally contains ceramide, diacylglycerol, cholesterol and various transmembrane (surface) proteins, such as tetraspanins (CD9, CD63, CD81, CD82), fusion and transferring proteins (Rab2, Rab7, flotillin and annexin), lysosome-associated membrane glycoproteins (LAMP1 and LAMP2), heat shock proteins (Hsc70 and Hsc90), the tumor-sensitive gene 101 (Tsg101), cytoskeleton proteins (actin, myosin and tubulin), integrins, transferrin receptors, and MHC class I and II molecules [16,32,36,37,38,39].

Exosomes have promising potential as drug and gene delivery vehicles, and can be used in tissue regeneration, immunomodulation, and as disease identifiers [15,18]. They are also crucial in the coagulation process, intercellular signaling, and cell waste management [40].

**Figure 2:**
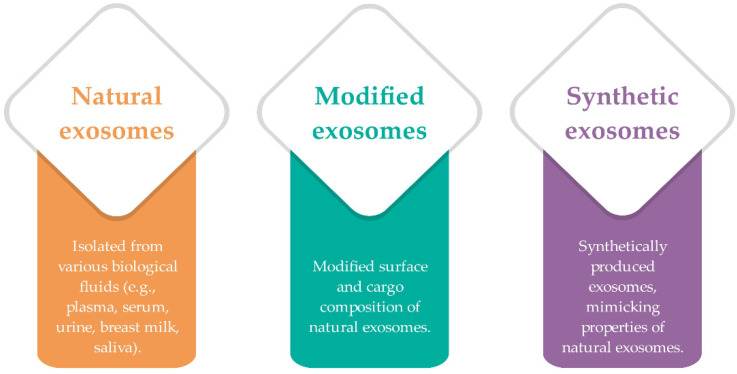
Schematic representation of exosomes classification according to their origin—natural (isolated from various biological fluids), modified (natural produced and modified for specific purposes), and synthetic exosomes (they mimic properties of natural exosomes; summarized from [16,18,20]).

Recently, a lot of attention has been paid to artificial exosomes, which are considered to be better potential therapeutic biomaterials than natural exosomes. Exosomes can be classified according to their origin, and therefore divided into natural, modified, and synthetic exosomes (Figure 2) [18].

### 2.1. Natural Exosomes

Exosomes are natural nanomaterials [41] secreted from different types of cells, including epithelial cells, endothelial cells, mesenchymal stem cells, macrophages, dendritic cells, tumor cells, neurons, oligodendrocytes, reticulocytes, mast cells, platelets and cancer cells, B and T cells, and astrocytes by exocytosis [16,37,40,42]. They are present in most body fluids, including plasma [43], serum [44,45], urine [46,47], breast milk [48,49,50], semen [51,52], saliva [53,54], nasal secretion [37], lymph [16], amniotic fluid [55,56], ascites [57,58], cerebrospinal fluid [59,60,61], etc. Exosomes are potential natural therapeutics, due to their biocompatibility [7].

#### 2.1.1. Exosomes Isolation Techniques

**Figure 3:**
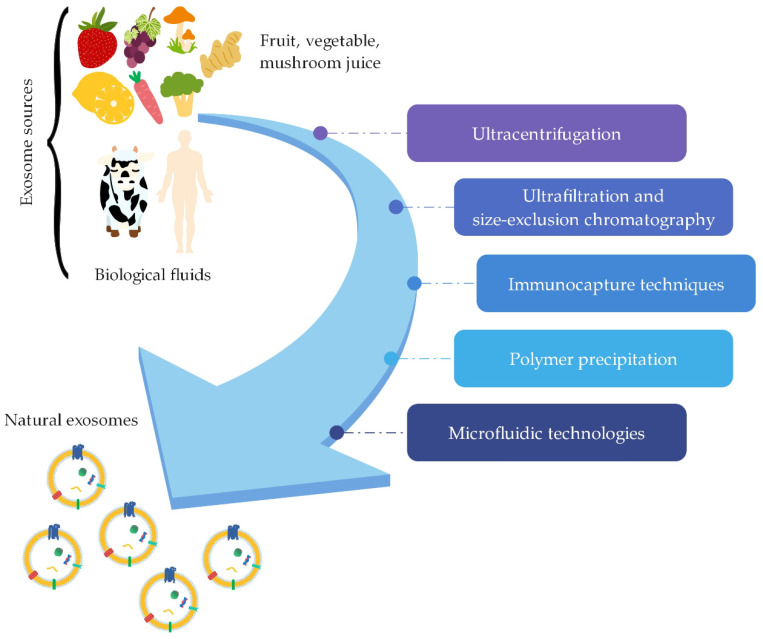
Various methods (ultracentrifugation, ultrafiltration and size-exclusion chromatography, immunocapture techniques, polymer precipitation, and microfluidic technologies) for natural exosomes isolation from different sources (e.g., biological fluids and fruit, vegetable, and mushroom juices; summarized from [8,11,16,62]).

Various methods (Figure 3) have been developed to successfully isolate exosomes from different sources. The most commonly used technique for isolating exosomes is ultracentrifugation, which provides high amounts of isolated exosomes [11,62,63]. This technique is based on the difference in density and particle size and is a simple and cost-effective method [8,64]. It involves differential ultracentrifugation and density-gradient ultracentrifugation [16]. Ultrafiltration and size-exclusion chromatography are isolation methods based on the separation of biomolecules according to their size [11,62,64,65]. Methods based on interactions between antibodies and proteins on the surface of exosomes for exosome isolation are immunocapture techniques [16,21,62]. Another method for isolation of exosomes is polymer precipitation [66], an easy and simple method [62] based on changing their solubility [16]. Microfluidic technologies are also being used to isolate and purify exosomes. These are improved methods with high purity and sensitivity [16,62,64].

#### 2.1.2. Natural Exosome-like Nanoparticles

Natural exosome-like nanoparticles can be classified into exosomes derived from animals and exosomes derived from plants [11]. Animal exosomes are produced primarily from immune cells (lymphocytes, red blood cells, platelets, dendritic cells, tumor cells) and are present in various biofluids (urine, milk, plasma). The most researched exosomes of animal origin are exosomes isolated from bovine milk [16]. Recently, increasing attention is being paid to exosome-like nanoparticles derived from plants (plant exosomes) [8], with a comparable structure to animal and human exosomes [11]. However, they differ from these in the composition of proteins, lipids, and RNA [67].

Exosome-like nanoparticles isolated from a variety of plant sources have the potential to be used as therapeutic drug delivery vehicles for the treatment of certain diseases [67,68]. Edible plant exosomes derived from ginger, lemon, grapefruit, grape, broccoli, and carrot would be suitable for the treatment of inflammatory diseases due to their anti-inflammatory properties [69,70,71,72,73]. Perut et al. [74] isolated and purified exosomes from strawberries that had a similar morphology to mammalian exosomes. Exosomes derived from strawberry juice have been found to prevent oxidative stress and are non-toxic.

Exosome-like nanoparticles are also naturally present in mushrooms and contain lipids, proteins, and RNA. Liu et al. [75] successfully isolated exosomes from different edible mushrooms (*Hypsizygus tessellatus*, *Agaricus bisporus*, *Pleurotus eryngii*, *Lentinula edodes*, and *Pleurotus ostreatus*) by sequential centrifugation. Among these, exosomes isolated from shiitake mushroom (*L. edodes*) showed strong anti-inflammatory activity and potential for the treatment of fulminant hepatic failure (FHF).

The most commonly used isolation technique for isolating animal-derived and plant-derived exosomes is differential ultracentrifugation [8]. Other techniques used to isolate the aforementioned exosomes are ultrafiltration, size exclusion chromatography, precipitation, and microfluidic technologies [67]. After isolation, various biologically active components can be incorporated into them and used as drug delivery vehicles [8].

### 2.2. Modified Exosomes

Naturally produced exosomes can be modified for specific therapeutic purposes [20], including the incorporation of drugs and other therapeutic agents, as well as changing the surface charge for faster drug uptake [18].

Exosomes produced from various natural sources, such as different fruit and vegetable juices and mammalian biological fluids, have already been modified in numerous studies in order to verify their potential for biomedical applications.

Exosomes can be modified in two different ways, by interior modification, where the structure of the cargo within the exosome is modified, and surface modification, where the structure of the outer surface of the exosome is modified. 

#### 2.2.1. Interior Modifications

**Figure 4:**
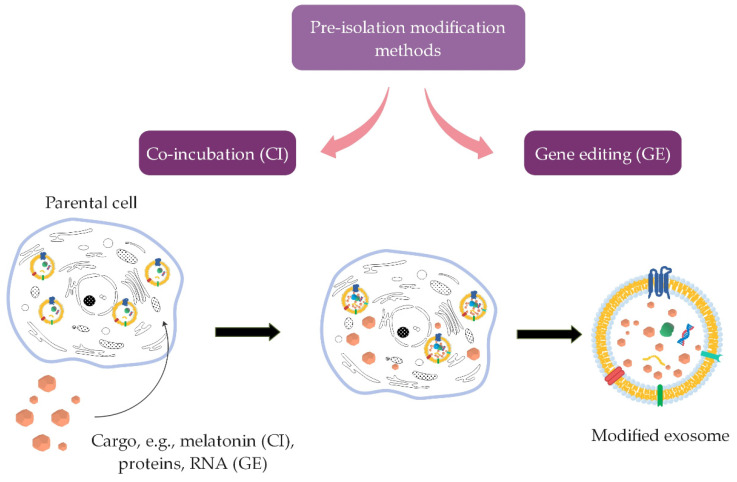
Principle of pre-isolation exosomes modification methods (co-incubation and gene editing (intended for the incorporation of RNA and proteins), where the modification of exosomes is performed before isolating exosomes from parental cells (summarized from [7,11,76]).

**Figure 5:**
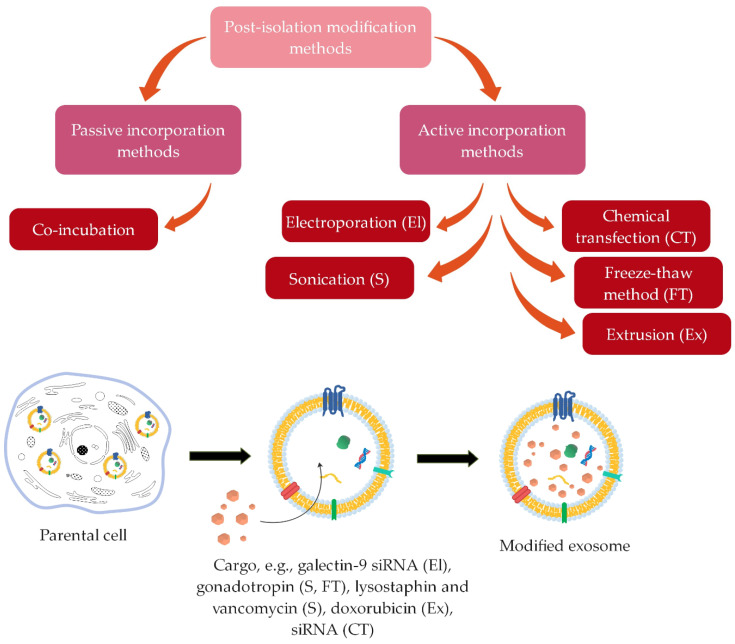
Principle of post-isolation exosomes modification methods, where the desired therapeutic agents can be encapsulated in purified exosomes, directly after their isolation from cells, either through passive (co-incubation) or active incorporation methods (summarized from [14,15,16,79,80,81,82,83,84]).

Interior modifications include methods for incorporating therapeutic agents into the interior of naturally derived exosomes. These methods can ensure different efficiency and stability of the incorporated cargo [7]. They are further divided into pre-isolation (Figure 4) and post-isolation modification methods (Figure 5) for incorporation of cargo, depending on whether the modifications are performed before or after exosome isolation.

##### Pre-Isolation Modification Methods

In pre-isolation modification methods, modification is performed prior to exosome isolation from cells (Figure 4).

The parental cells are modified by the method of incubation with the desired drug, whereby this drug is then encapsulated into the cells. From these modified cells, exosomes that already contain the incorporated desired drug are then secreted and isolated [7,15,16,77]. This is a relatively simple method, but it is not possible to provide control over loading efficiency [78]. This method was performed on mesenchymal stromal cells into which melatonin was incorporated. These cells then produced exosomes containing melatonin [76]. Another method is gene editing, where genetic modification of parental cells is used to incorporate therapeutic cargo such as RNA and proteins that cannot be directly incorporated into exosomes [11].

##### Post-Isolation Modification Methods

Drugs and therapeutic agents can be encapsulated into purified exosomes by post-isolation modification methods directly after their isolation from cells, which provides greater efficiency (Figure 5).

This can be achieved through active or passive incorporation [15,16,85]. The passive incorporation methods are relatively simple and successful and preserve the morphology of exosomes but provide low loading efficiency. They include co-incubation of exosomes and therapeutic agents that can diffuse into the interior of exosomes through the membrane along the concentration gradient [14,85].

On the other hand, active incorporation methods involve different approaches for loading therapeutic agents into exosomes [14]. These methods temporarily disrupt the membrane, allowing the cargo to easily pass into the interior of the exosomes. After the diffusion of the cargo, the membrane integrity of the exosomes is restored [8]. One of the active incorporation methods is electroporation, in which pores are temporarily formed in the phospholipid bilayer of exosomes due to the electric field in a conductive solution, allowing the entry of cargo into exosomes [14,16,86]. Faruqu et al. [79] incorporated fluorescent Atto655-conjugated nonspecific siRNA into exosomes derived from human embryonic kidney cells (HEK-293 cells) by electroporation with 10–20% efficiency. Zhou et al. [83] also successfully encapsulated galectin-9 siRNA into exosomes derived from bone marrow mesenchymal stem cells (BM-MSCs) with the use of a Gene Pulser X Cell Electroporation System. In the sonication process, the membrane is deformed using ultrasound and a homogenization probe, thus allowing the drug to diffuse into exosomes [14,85]. Human chorionic gonadotropin was efficiently loaded (40.55% ± 4.21%) into exosomes isolated from uterine fluid using a sonication process by Hajipour et al. [80]. While Yang et al. [84] achieved 15.52% ± 2.38% encapsulation efficiency by encapsulating the antibacterial drug lysostaphin into mannosylated exosomes by sonication and 22.15% ± 3.21% by encapsulating vancomycin. Extrusion is a method in which a mixture of exosomes and cargo is extruded through a membrane with a pore size between 100 and 400 nm using a lipid extruder. The cargo enters inside the exosomes through a disrupted membrane [16]. Guo et al. [82] encapsulated doxorubicin into exosomes by a magnetic extrusion process. With the ammonium sulfate gradient loading mechanism, they achieved much higher encapsulation efficiency (68%) than with direct encapsulation (23%). In the freeze–thaw method, several cycles of freezing the exosome-cargo mixture at −80 °C or in liquid nitrogen and re-thawing to room temperature are repeated to ensure the successful incorporation of drugs [16,86]. Hajipour et al. [80] incorporated human chorionic gonadotropin into exosomes from RAW264.7 cells with 14.02 ± 5.46% efficiency using the freeze–thaw method. Another method is chemical transfection, in which exosomes and cargo are incubated with the surfactant, causing the formation of pores in the membrane and thus the penetration of drugs. The most frequently used surfactant is saponin, and thus this method is also called saponin-assisted loading [14,78]. Warren et al. [81] established that encapsulation of siRNA into bovine milk-derived exosomes by chemical transfection was significantly more efficient than by electroporation. 

#### 2.2.2. Surface Modifications

The surface of exosomes is essential for their biodistribution, ability to target specific cells, and therapeutic potential. By modifying the surface the desired characteristics of exosomes can be achieved, thereby improving cell targeting [14,85,87]. The exosome’s surface can be modified through acting on parental cells that will secrete exosomes or through directly modifying isolated exosomes [16].

##### Genetic Engineering of Parental Cells

**Figure 6:**
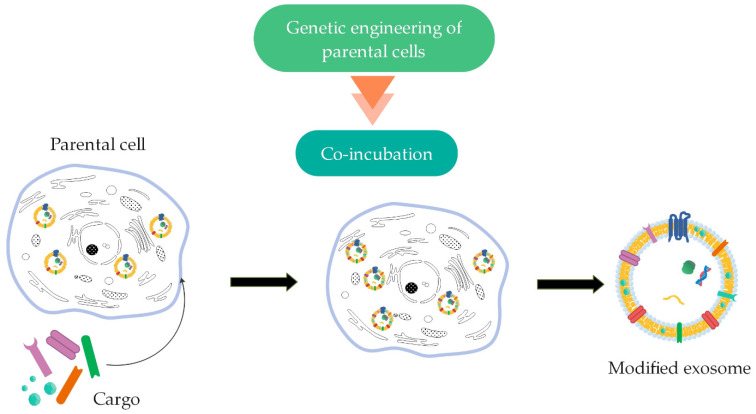
Genetic engineering of parental cells for surface modification of exosomes by the method of co-incubation (summarized from [11,16]).

Modification of the exosome membrane can be obtained through genetic engineering of parental cells [11] (Figure 6). Cells are genetically modified through viral vectors by inserting the coding sequence of the desired ligand. These cells then secrete exosomes with expressed peptides on their surface [14,16,88].

##### Direct Modification of Isolated Exosomes

**Figure 7:**
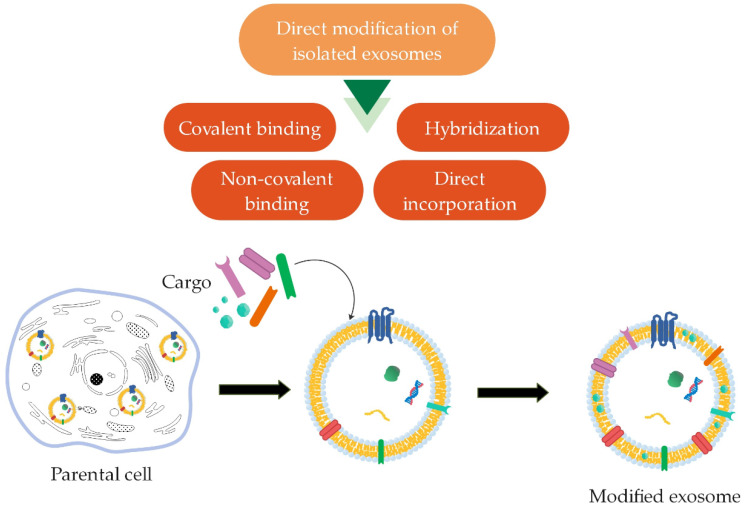
Direct surface modification of isolated exosomes using different methods (covalent binding, non-covalent binding, hybridization, and direct incorporation; summarized from [16,85]).

Certain methods have been developed to modify the surface of exosomes after their isolation from cells (Figure 7) in order to achieve a more specific delivery to the target cells [14]. Surface modification of exosomes due to covalent binding can be performed through a crosslinking reaction called click chemistry or azide-alkyne cycloaddition. A reaction between an alkyl and an azide chemical group occurs to form a stable triazole bond [14,16,89]. Using this method, Tian et al. [90] modified the surface of exosomes derived from mesenchymal stem cells (MSCs) with a cyclo(Arg-Gly-Asp-D-Tyr-Lys) peptide [c(RGDyK)] in order to improve targeting abilities. For the same purpose, Xu et al. [91] fluorescently labeled exosomes from pancreatic cells with a newly developed method based on copper-free click chemistry. However, the surface can also be altered through various non-covalent modification methods. The most commonly used modifications are the receptor-ligand binding method and a multivalent electrostatic approach based on interactions between highly cationic species and negatively charged functional groups on the membrane [16,89].

One of the methods of surface modification is hybridization, wherein exosomes combine with fusogenic liposomes due to the lipid nature of exosomes’ membrane. Moreover, due to the exosomes’ lipid membrane, hydrophobic components can be incorporated directly onto their surface [85].

### 2.3. Synthetic Exosomes

**Figure 8:**
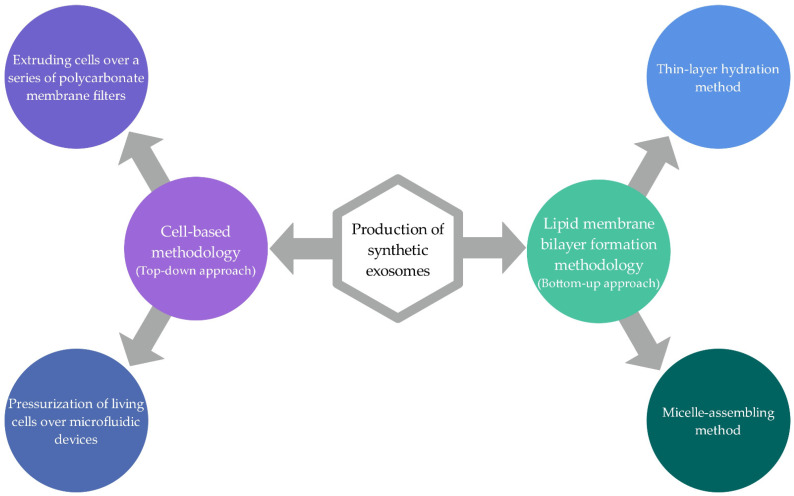
Schematic representation of methodologies for the production of synthetic exosomes—cell-based methodology and lipid membrane bilayer formation methodology (summarized from [9,20]).

For the possibility of using modified exosomes for a wide range of therapeutic applications, it is necessary to provide standardized isolation and purification with the appropriate clinical grade of natural exosomes, which is difficult to achieve. Further, suitable modification techniques for incorporation of drugs, genes, and other therapeutic agents, for which not all developed approaches are fully appropriate, also have to be provided. As a result, approaches are being developed to produce completely artificial exosomes using biotechnology that mimic the properties of exosomes. However, there are still not many studies covering the field of synthetic exosomes [20,92]. Two approaches (Figure 8) have been developed for synthetic exosome production, cell-based methodology, and lipid membrane bilayer formation methodology.

#### 2.3.1. Cell-Based Methodology

The cell-based methodology is based on top-down technology, which is used to fabricate smaller materials from large and complex substrates. In this, cultured cells are used as a basis for the production of synthetic biomaterials, which are broken down into smaller membrane fragments. These fragments assemble themselves into spherical membrane vesicles that carry the same membrane characteristics as the initial cell [9,14,18,20]. According to the principle of top-down methodology, exosomes can be produced by different approaches, among which there are two most appropriate and promising methods for producing larger amounts of therapeutic biopolymers similar to naturally isolated exosomes. The first, simpler approach is the process of extruding cells over a series of polycarbonate membrane filters with reduced pore size, producing vesicles of a similar size [9,14]. The second approach involves the pressurization of living cells over microfluidic devices, which contain a series of parallel hydrophilic microchannels, whereby the cells are broken down into smaller fragments and then reassembled into vesicles [9,20].

#### 2.3.2. Lipid Membrane Bilayer Formation Methodology

In contrast to cell-based methodology, lipid membrane bilayer formation is performed according to the principle of bottom-up methodology, which is based on the production of larger and more complex structures from small components [14,20]. For the production of therapeutic biomaterials, special lipids required for the production of the lipid bilayer, specific membrane proteins, and the desired therapeutic components (cargoes) are used as molecular building blocks [18]. Exosomes are structurally and biochemically similar to liposomes, and therefore according to the principle of bottom-up techniques, two main approaches are suitable for the fabrication of exosomes, among various methods for the production of liposomes [14,18]. This is the thin-film hydration method based on the hydration of a dried film and the microemulsion and micelle assembling method [14,20,93]. Some other methods potentially suitable to produce vesicles similar to natural exosomes are reverse-phase evaporation, a method based on ethanol and ether injection, microfluidic-based methods, extrusion methods, and homogenization techniques [16,18]. During these production processes, the desired cargo can also be incorporated [9].

Zhang et al. [92] used a combination of bottom-up and top-down approaches in their study to produce artificial chimeric exosomes for anti-phagocytosis and targeted cancer therapy. Exosomes were constructed on the principle of incorporating membrane proteins from different cell types, including red blood cells and MCF-7 cancer cells, into a synthetic phospholipid bilayer. With this approach they were able to closely mimick the morphological and physiological composition of natural exosomes, as well as the anti-tumor therapeutic effect, as shown with a study of mice with subcutaneous injection.

### 2.4. Advantages and Disadvantages of the Individual Exosome Preparation Method

A comparison of the previously mentioned preparation and production methods of modified and synthetic exosomes is presented below based on their advantages and disadvantages.

#### 2.4.1. Comparison of Modification Methods of Exosome Preparation

**Figure 9:**
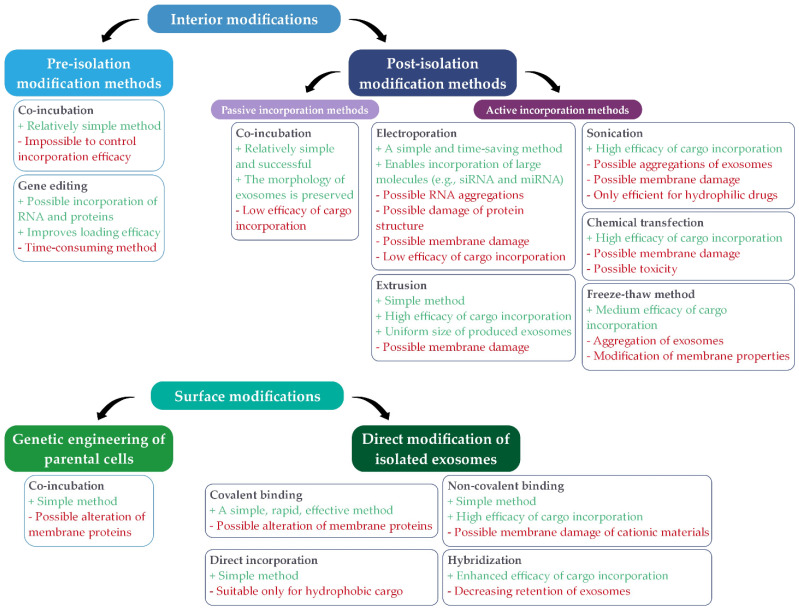
The main advantages (green) and disadvantages (red) of exosome preparation methods for interior (pre-isolation and post-isolation modification methods) and surface modifications (genetic engineering of parental cells and direct modification of isolated exosomes; summarized from [7,11,14,16,78,85,89,90,94,95,96]).

Figure 9 summarizes the advantages (green) and disadvantages (red) of modification methods of exosome preparation. These methods are divided into interior and surface modifications (passive and active incorporation methods).

The simplest method for producing modified exosomes is co-incubation, which can be used as an internal (pre-isolation and post-isolation methods) as well as a surface modification technique. The disadvantage of this method is usually the low efficacy of cargo incorporation. The method of incorporating therapeutic agents into the interior of exosomes directly after their isolation from cells is more successful. Electroporation and extrusion are also simple methods, as are almost all methods of direct modification of isolated exosomes, i.e., covalent binding, non-covalent binding, and direct incorporation. Among the latter, covalent binding is also a rapid and efficient method [7,11,14,16,85].

The efficiency of cargo incorporation into the interior of exosomes is extremely high in the case of sonication, chemical transfection, and extrusion. However, the mentioned methods also have some disadvantages that play a crucial role in choosing a method for cargo loading inside exosomes. In particular, they can cause deformation of the membrane of exosomes, while chemical transfection also has possible toxicity to living cells. In contrast, the incorporation in the freeze–thaw method is only moderately effective and low in the co-incubation method. Although electroporation does not have a high efficiency of cargo incorporation, it is suitable for the incorporation of large components into exosomes, such as siRNA and miRNA, but RNA aggregation is possible. In the case of surface modifications, non-covalent binding and hybridization are methods with high efficiency of incorporating cargo onto the exosomes’ surface. Most of the preparation methods of modified exosomes can lead to membrane damage or alteration of surface proteins. The only exception is co-incubation in internal modifications as well as hybridization and direct incorporation in surface modifications [14,16,85,89,94,95,96].

Some methods are only suitable for loading hydrophilic cargo, such as sonication (incorporation of cargo into the hydrophilic interior of exosomes), while others are only suitable for loading hydrophobic cargo, such as direct incorporation (incorporation of cargo onto exosomes hydrophobic surface) [85,95].

Due to the possible incorporation of RNA and proteins without destroying the structure of RNA, gene editing is a highly suitable method for biomedical applications, especially for gene delivery. However, it is a time-consuming method [11,95].

As presented above, there is no most ideal method of exosome preparation. Each method has certain advantages or disadvantages. Therefore, the choice of the exosome production method itself depends on various requirements, such as the type and size of molecules for incorporation, simplicity of the method, available equipment, loading efficiency, etc.

#### 2.4.2. Comparison of Methodologies for Synthetic Exosome Production

Table 1 presents the advantages and disadvantages of methodologies for synthetic exosome production, with both cell-based and lipid membrane bilayer formation methods.

Compared with the preparation of modified exosomes, synthetic exosome production has some advantages. Extremely large amounts of exosomes can be obtained with cell-based methods, while extremely pure products with a known composition can be produced using the lipidmembrane bilayer formation methods [9,14,20,85].

Given the different advantages and disadvantages of both approaches for synthetic exosome production, it cannot be confirmed which method is optimal. The production approach depends on how pure a product or how much of a product one wants to obtain, what specifications are required, and the primary purpose of their use. Nevertheless, they also have key disadvantages as they are more expensive or time-consuming methods, and there is a great need for deep knowledge of the composition of exosomes.

### 2.5. Incorporation of Cargo into Therapeutic Biomaterials

**Figure 10:**
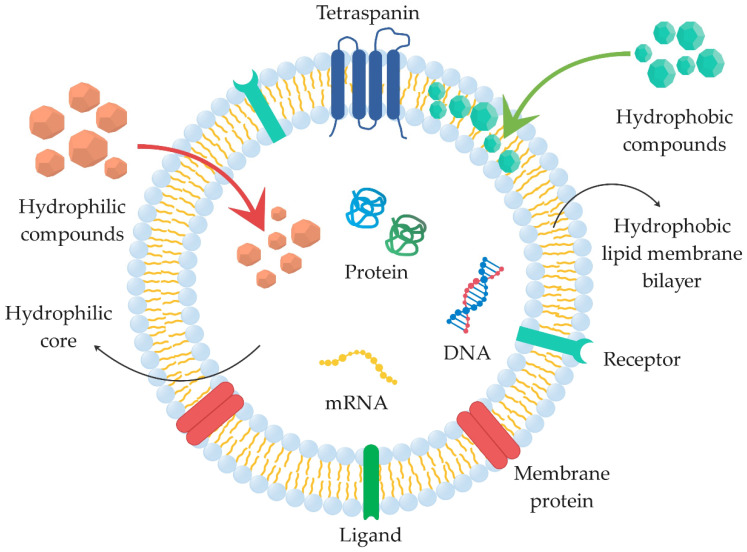
Incorporation of hydrophilic (e.g., melatonin, gemcitabine, linezolid, vancomycin, and lysostaphin) and hydrophobic (e.g., curcumin, paclitaxel, doxorubicin, and aspirin) cargo into hydrophilic core and hydrophobic lipid membrane bilayer of exosomes (summarized from [7,76,84,85,100,102,104,105,106,107,108,109,110]).

Exosomes consist of a hydrophobic lipid membrane bilayer and a hydrophilic core (Figure 10). Due to surface modification in order to improve exosome imaging and cell targeting, various hydrophobic therapeutic components (e.g., paclitaxel and curcumin) can be incorporated into the lipid membrane bilayer. This can improve the stability and efficacy of the incorporated drugs. In contrast, numerous hydrophilic therapeutic cargoes, including hydrophilic drugs and macromolecules, such as RNA, DNA, and proteins, can be incorporated into the core of exosomes and thereby improve cell delivery [96,99,100,101].

Hydrophilic compounds are not able to pass naturally through the lipid bilayer; therefore, different methods have been developed to incorporate various compounds into exosomes. These methods create pores through which hydrophilic compounds can enter into exosomes [102], and are briefly described in Section 2.2.1. For the incorporation of hydrophobic compounds, the method of co-incubation of exosomes with hydrophobic therapeutic agents alone is sufficient, as they can easily pass into the membrane [102]. However, this method is only recommended for smaller hydrophobic molecules, and therefore other modification methods are also used for the incorporation of lipophilic molecules [103].

Various methods such as co-incubation, saponin-assisted loading, the freeze–thaw method, sonication, and extrusion can effectively incorporate proteins, especially the enzyme catalase, without altering the structure of the exosomes significantly. In addition, due to the preserved enzymatic activity of catalase, such modified exosomes effectively reduce oxidative stress and produce a strong neuroprotective effect, representing potential use in the treatment of inflammation, stroke, and neurodegenerative diseases, particularly Parkinson’s disease and Alzheimer’s disease, and infectious diseases, such as meningitis, encephalitis, and neurocognitive disorders in HIV-infected individuals [111]. Exosomes modified with therapeutic peptides and hydrophobic components such as curcumin can successfully reduce inflammation in the lungs. Therefore, such modified exosomes could be useful in the treatment of fatal respiratory diseases, including acute lung injury [112]. Lipophilic and water-insoluble components are more difficult to deliver to the target site and thus ensure adequate therapeutic efficacy. Exosomes have become potential carriers of these components due to their lipid bilayer membrane, into which hydrophobic therapeutics can be incorporated, thereby their importance for biomedical applications has increased. In addition, exosomes with the incorporated hydrophobic anticancer drug may increase the cytotoxicity of the drug, which may lead to the development of safe and improved cancer therapy [113]. Table 2 presents some examples of incorporated hydrophobic and hydrophilic components into exosomes by different methods from recent studies from the last three years.

Nanovesicles produced from a variety of plant sources have similar properties to mammalian exosomes. Therefore, just as various therapeutic agents can be incorporated into mammalian exosomes, different therapeutic cargo, such as small molecular drugs, siRNAs, DNA expression vectors, and proteins [70], can also be incorporated into plant exosomes, as shown in Table 3, which includes some examples from studies from the last six years. Moreover, edible plant exosomes are non-toxic and can be produced on a large scale [120].

In the next section, various promising applications of therapeutic exosomes are presented, with an emphasis on the delivery of drugs and other therapeutic agents.

## 3. Therapeutic Applications of Exosomes

**Figure 11:**
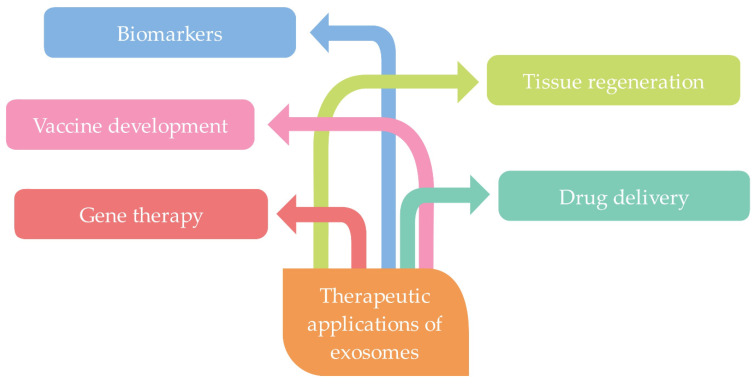
Schematic representation of various therapeutic applications (e.g., gene therapy, vaccine development, tissue regeneration, drug delivery, and as biomarkers in the diagnosis and therapy) of exosomes (summarized from [15,21]).

Due to the aforementioned properties, exosomes obtained from various sources and modified by different processes or synthetically produced can be used for a variety of biomedical applications (Figure 11) [15]. They can be used in drug delivery, gene therapy, vaccine development, tissue regeneration, and as biomarkers in the diagnosis and therapy of various diseases, such as cardiovascular diseases, cancer, neurodegenerative diseases, skin regeneration, arthritis, diabetes, and for immunological purposes [15,21,127,128,129].

Exosomes are stable therapeutic biomaterials, even in digestive and other biological fluids, and are therefore highly effective for long-distance intracellular communication. They also possess a natural targeting ability due to their unique surface composition. However, their targeting ability to recipient cells varies according to their origin. In order to make exosomes easier for the receiving cells to recognize, certain molecules can be incorporated into them. Exosomes can offload their cargo into target cells through membrane fusion or phagocytosis [8].

### 3.1. Biomarkers

Biomarkers are important for the early detection of disease and effective therapy. They must be specific, noninvasive, and have high stability. As there is currently a lack of such biomarkers, more attention is being paid to exosomes and their content as promising biomarkers [130,131,132,133]. Exosomes can be isolated from various body fluids and contain components of the cell from which they are secreted, and therefore are easily accessible for diagnosis and studying complex diseases, as they act as a fingerprint of parental cells [134] and reflect their pathological status [135]. Exosomes are already being used as biomarkers, most commonly for the diagnosis of cancer, as well as for cardiovascular diseases, central nervous system disorders, and infectious diseases [11,127,136], and are sensitive and reliable [137].

Jia et al. [138] determined in their study that exosome proteins derived from neurons (GAP43, neurogranin, SNAP25, and synaptotagmin 1) have diagnostic potential for Alzheimer’s disease and amnestic mild cognitive impairment. In addition, a combination of exosomal synaptic proteins can predict Alzheimer’s disease approximately 5–7 years before cognitive impairment. Among others, exosomes play a significant role in the regulation of skin homeostasis, as well as in skin regeneration and as therapeutics and biomarkers in various dermatological diseases, including melanoma, Merkel cell carcinoma, cutaneous pigmentation, and psoriasis [29].

### 3.2. Vaccine Development

Exosomes have many potential applications for biomedical purposes and, among others, can also be used as vaccination vectors. Recently, much attention has been paid to the possibility of their use as cell-free vaccines against cancer and in immune therapy [139,140,141,142,143]. On the other hand, exosomes also play an important role in the design of vaccines against various infectious diseases, as they can modulate immune responses. They could especially be used in combatting bacterial infections, as they could potentially identify and kill pathogens [144]. They also have the potential to provide an effective approach for the development of virus-free vaccines due to their ability to deliver antigens to target cells. Based on this and on various properties such as anti-inflammatory, proangiogenic, and immunomodulatory activity of exosomes, much attention is currently being paid to exploring the potential for immunomodulatory treatment in patients infected with SARS-CoV-2, especially exosomes derived from MSCs [145,146].

### 3.3. Gene Therapy

Gene therapy does not use a therapeutic approach to alleviate symptoms as in conventional treatment therapy, but this therapy is aimed at the complete cure or elimination of the disease. It is especially important in individuals with congenital genetic disorders. Because bio-vectors currently used in gene therapy can have several adverse side effects in individuals, and because they must be non-toxic while providing a high level of efficiency, exosomes as bio-vectors have become increasingly important in this field as well [147,148]. Stem cell exosomes have many advantages over other vectors in gene therapy in the treatment of cardiovascular diseases. The most important advantage is that their membrane can effectively protect the cargo (RNA/gene) from digestion during delivery, and that target cells can take them up quickly and efficiently. Therefore, exosomes can be highly efficient carriers in gene delivery [149]. Li et al. [150] demonstrated that exosomes are adequate vectors in the treatment of familial hypercholesterolemia, which may lead to atherosclerosis and cardiovascular diseases. The incorporated mRNA in exosomes was stable and successfully translated into functional proteins in target cells, resulting in reduced lipid deposition in the liver and reduced serum LDL-cholesterol levels. Vakhsiteh et al. [151] used XMIRXpress-34a lentivectors to genetically modify dental pulp MSCs (DPSCs) with tumor suppressor miR-34a, from which exosomes with incorporated miR-34a were then isolated, which showed anticancer effects on breast cancer cells in vitro.

### 3.4. Tissue Regeneration

It has been shown that various complications can occur in traditional tissue engineering, and the efficiency of regeneration itself can be relatively low. Therefore, due to their good characteristics, minimal side effects, and potentially good efficacy, exosomes have also begun to be used in this field [152,153,154].

In tissue engineering, MSCs are primarily used to fabricate and induce the complete replacement of damaged functional tissues or organs. Various studies have shown that exosomes secreted from MSCs are important in the regeneration of bones, muscles, blood vessels and nerves, cartilage, dentin, as well as in oral and craniofacial regeneration. By modifying exosomes, nanoparticles with adequate characteristics for successful tissue regeneration can be created [155]. Li et al. [156] constructed a cell-free tissue-engineered system by combining exosomes derived from adipose-derived stem cells (hASCs) with poly(lactic-co-glycolic acid) scaffolds with a polydopamine coating (PLGA/pDA). These scaffolds have successfully accelerated bone regeneration in critical-sized calvarial bone defects in mice.

### 3.5. Delivery of Drugs and Therapeutic Agents

Due to their specific characteristics, exosomes could be efficiently used as vehicles for the delivery of drugs and other therapeutic agents, particularly exosomes secreted from MSCs and tumor cells, due to their therapeutic potential, and from immune cells, from which exosomes can be economically obtained on a large scale [9,157]. Many studies have already confirmed the use of exosomes as potentially effective nanocarriers for drugs and therapeutic agents [66,127,158,159,160].

Exosomes with encapsulated curcumin have been shown to increase the solubility, stability, and bioavailability of curcumin, as compared with free curcumin, as well as increased drug penetration through the blood–brain barrier, which is difficult to achieve with conventional drugs. In addition, curcumin-encapsulated exosomes have great potential in the treatment of Alzheimer’s disease, as a study on mice showed improved cognitive functions [161]. Similarly, exosomes incorporated with chemotherapeutic agent Paclitaxel show greater cytotoxicity to glioblastoma multiforme cells than Paclitaxel alone [105]. Exosomes isolated from MSCs with incorporated melatonin compared with exosomes without incorporated melatonin showed better therapeutic and protective properties in renal damage caused by renal ischemia-reperfusion injury [76].

These therapeutic nanocarriers are promising in the treatment of breast cancer as they improve the effectiveness of therapeutic agents [162,163]. They are being developed as important drug suppliers, primarily for the treatment of chemotherapy-resistant patients [164]. Macrophage-derived exosomes can carry miRNA, thereby regulating resistance to chemotherapy. Li et al. [165] achieved highly effective targeted chemotherapy of triple-negative breast cancer using macrophage-derived exosomes with incorporated poly(lactic-co-glycolic acid) (PLGA) nanoparticles loaded with doxorubicin and with an additional modified surface with a peptide for improved tumor targeting efficiency. This also enhanced the cellular uptake and antitumoral efficacy of the loaded drug, as well as increased growth inhibition and cell death of tumor cells. Exosomes with an encapsulated adequate antibiotic or other therapeutic agent may help treat intracellular infections caused by pathogenic microorganisms, as they represent a safe, successful, and cost-effective method. Yang et al. [110] formulated nanovesicles to overcome methicillin-resistant *Staphylococcus aureus* (MRSA)-induced infection. The synthetic antibiotic linezolid was incorporated into exosomes produced from mouse RAW264.7 macrophages. The use of exosomes with incorporated linezolid has been shown to be a more effective therapeutic method in the treatment of MRSA infections, both in vivo and in vitro, in comparison with the administration of free linezolid. It was also determined that the prepared therapeutic biomaterials did not cause cytotoxicity in macrophages.

Exosomes with or without modification are particularly ideal in the delivery of drugs and other therapeutic agents, which can be introduced into the body through different administration routes.

**Figure 12:**
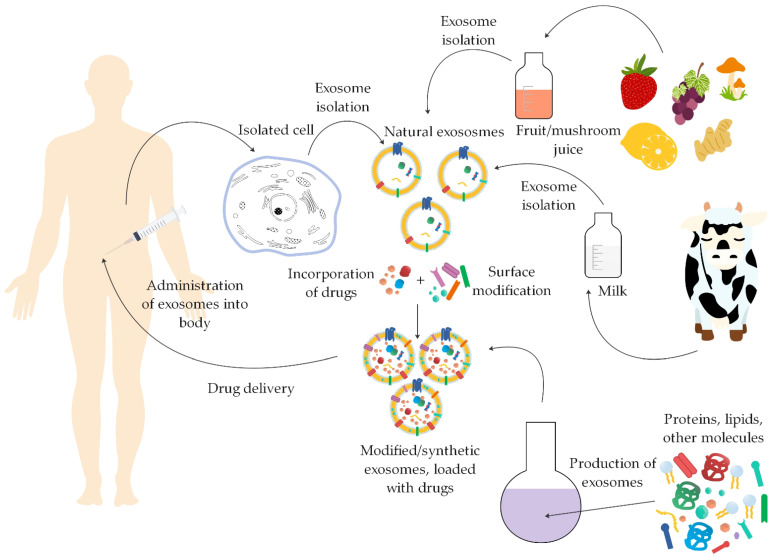
The pathway of production (synthetic or modified) or isolation (natural) of exosomes for use as drug delivery vehicles through various routes of administration into the body (summarized from [14,16,18,20,74,75,85,122,125]).

Figure 12 shows the pathway of the production of exosomes for use as drug carriers.

## 4. Drug Delivery of Therapeutic Biomaterials through Different Administration Routes

Compared with the conventional administration of drugs in free form, drug delivery via nanocarriers is becoming an increasingly important system as it improves treatment efficacy. The use of conventional therapies can also damage healthy cells [166].

For successful drug delivery, drug delivery vehicles must meet certain criteria. It is necessary that an adequate amount of the drug can be incorporated into drug carriers and at the same time ensure the specific delivery of drugs and appropriate therapeutic effect. They must be non-toxic or with extremely low toxicity and biocompatible with the response of the immune system to prevent their degradation before they reach their targets. Many exosomes have these characteristics. In addition, they are small, have extremely low toxicity, and can cause a low rate of long-term accumulation in organs and tissues [66,127,167]. They can also cross various biological fluids and pass through plasma membranes, thus delivering therapeutic compounds into the cytoplasm of target cells [147]. Therefore, exosomes have high potential as delivery vehicles of therapeutic agents, particularly because they can also cross the blood–brain barrier [168,169]. Compared with other drug carriers, due to the abovementioned properties, they have a greater potential to be used for biomedical purposes, especially in the treatment of more difficult to treat diseases, including cancer, neurodegenerative diseases, and cardiovascular diseases [139]. One of the advantages is also that exosomes can be utilized for the development of cell-free therapeutics that are safer than cell therapy [170,171]. For effective delivery of therapeutic agents, they need to be successfully incorporated into exosomes [172]. 

Drug delivery through therapeutic biomaterials, such as exosomes, is generally considered as a safe method. However, after dosing, immune responses are possible to occur since exosomes produced from human cells are mainly used, and this can lead to immunogenicity and toxicity, as well as increased clearance of exosomes from the body. On the other hand, exosomes are still safer compared with other synthetic drug nanocarriers. However, the choice of cells used to isolate the desired exosomes is highly important. [102].

**Figure 13:**
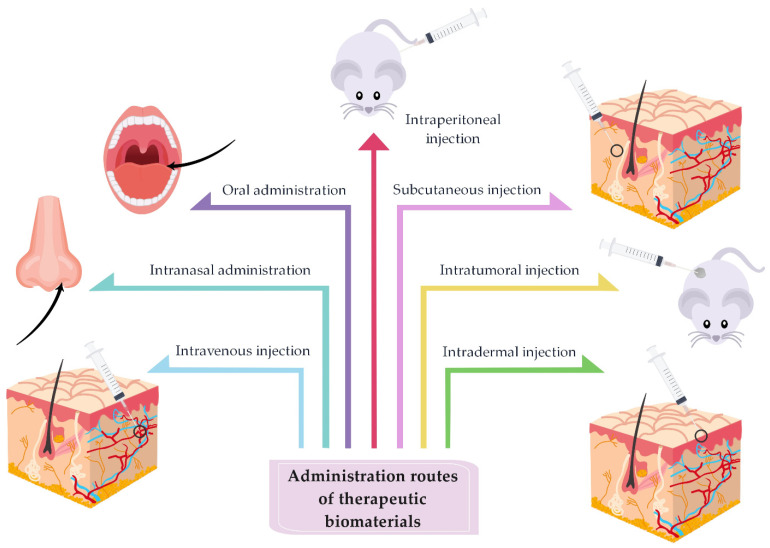
Representative structure of different administration routes of therapeutic biomaterials into the body through drug delivery: intravenous injection, intranasal administration, oral administration, intraperitoneal injection, subcutaneous injection, intratumoral injection, and intradermal injection (summarized from [2,11,102,147,173]).

The route of drug administration into the body and dosage are also significant in drug delivery. There are various routes of administration of therapeutic biomaterials, such as intravenous injection, subcutaneous injection, intraperitoneal injection, intratumoral injection, intranasal administration, oral administration, and intradermal administration. The route of administration of therapeutic biomaterials affects their distribution in tissues. Among all the mentioned routes, the intravenous route of therapeutics administration is the most commonly used and researched [2,11,102,147,173]. A brief description of the abovementioned administration routes of therapeutic biomaterials into the body through drug delivery (Figure 13), based on various studies performed in animal models, is presented below.

### 4.1. Drug Delivery through Intravenous Injection 

Intravenous injection, administration of drugs into the vein, is the most common route of administration, although it can lead to the accumulation of exosomes in the liver, spleen, and lung, and they can also be cleared from the circulation extremely quickly [173].

Qu et al. [174] determined that exosomes produced from mouse blood reticulocytes and loaded with dopamine have better therapeutic abilities in the treatment of Parkinson’s disease than compared with free dopamine. Dopamine-incorporated exosomes showed the ability to cross the blood–brain barrier, as well as lower systemic toxicity when administrated intravenously to mice. Intravenous or subcutaneous injection of exosomes from human adipose MSCs into mice resulted in improvement in atopic dermatitis [175].

### 4.2. Drug Delivery through Oral Administration

Therapeutic biomaterials do not accumulate in the liver to the same extent as compared with intravenous injection with oral administration. In addition, exosomes remain stable throughout the gastrointestinal tract due to their specific characteristics [176]. Exosomes derived from bovine milk and with incorporated Paclitaxel showed remarkable inhibitory properties on tumor growth in a mouse study compared with the same dose of Paclitaxel administrated intraperitoneally. In addition, they showed significantly lower systemic and immunogenic toxicity compared with intravenous injection [177,178].

### 4.3. Drug Delivery through Intranasal Administration

For the delivery of drugs to the central nervous system, the most effective way is through intranasal administration, that is, into the nose, as this avoids delivery through the blood–brain barrier [176]. Perets et al. [179] demonstrated that symptoms of autism spectrum disorders (ASD) were reduced in mice with intranasal administration of exosomes secreted from human BM-MSCs. There was an improvement in mutual interaction and a reduction in repetitive behavior, and no adverse effects were observed. Thus, exosomes may be used to treat ASD symptoms. Exosomes from MSCs have a neuroprotective effect, as they prevented perinatal brain injury through intranasal administration in mice [180].

### 4.4. Drug Delivery through Subcutaneous Injection

Subcutaneous injection, administrated beneath the skin, is effective in cutaneous malignancies and wound healing [173]. To treat MRSA infection, Yang et al. [110] used subcutaneous injection to deliver exosomes with an incorporated synthetic antibiotic to infected cells, and this proved to be an effective administration route. Gyeonghui et al. [181] injected exosomes obtained from different sources (RAW264.7 macrophage cell line, human serum, and fetal bovine serum) through subcutaneous administration into mice. Serum-derived exosomes incorporated with immune-stimulating biomolecules, such as CpG oligodeoxynucleotides (CpG ODN) and monophosphoryl lipid A (MPLA), have been shown to have exceptional properties as drug and immune stimulator carriers to the lymph nodes. After subcutaneous administration of exosomes with incorporated MLPA, activation and differentiation of T cells occurred, thereby increasing the cytokine IFN-γ and TNF-α induction for CD3+T cells. Therefore, MLPA-incorporated exosomes are significantly influential in achieving the desired immune responses.

### 4.5. Drug Delivery through Intratumoral Injection

In intratumoral administration, exosomes with incorporated drugs or therapeutic agents are injected directly into tumors. This causes degenerative changes in the tumor cells, thereby effectively reducing the size of the tumor [147]. By intratumoral administration of exosomes containing natural hyaluronidase PH20 and incorporated chemotherapeutic doxorubicin in mice with prostate cancer, effective inhibition of tumor growth due to increased exosome penetration and drug diffusion was achieved [182].

### 4.6. Drug Delivery through Intradermal Administration

Another one of the routes of exosome administration into the body is intradermal injection, which is achieved by injecting exosomes into the dermis. Morishita et al. [183] isolated exosomes from genetically engineered murine melanoma B16BL6 tumor cells that express the fusion protein streptavidin and lactadherin. They were then further modified with biotinylated immunostimulatory CpG DNA by streptavidin-biotin interaction. The exosomes prepared in this way were injected intradermally into mice, and the results showed successful antitumor effects.

### 4.7. Drug Delivery through Intraperitoneal Injection

Different routes of administration result in a different distribution of therapeutic biomaterials. Therefore, for optimal delivery of drugs to different organs, the appropriate choice of administration route is important. For example, exosomes were successfully delivered to the liver, spleen, and lungs by intravenous injection. In contrast, exosomes were more dispersedly distributed by intraperitoneal injection and, in addition to the liver, spleen, and lungs, effectively reached visceral adipose tissue. As a result, the administration of exosomes through intraperitoneal injection, administrated within the peritoneal cavity, has the potential to be used in the treatment of obesity [184].

Different administration routes of therapeutic biomaterials, including their therapeutic effects, are shown in Table 4.

Based on the properties of exosomes and the many studies already conducted both in vitro and in vivo, exosomes have shown the increasing potential for the development of new therapeutics. Many companies are already developing these, as shown in the following section.

## 5. Commercial Therapeutic Biomaterials

Various studies on exosomes have shown that they are non-toxic, even after repeated injections [190,191,192]. Therefore, exosomes are promising nanocarriers in the development of new therapeutic approaches to drug delivery and other therapeutic agents, due to their unique characteristics. Consequently, scientists from around the world are increasingly researching this field, and many commercial companies have successfully established various exosome platforms and produced therapeutic exosomes that are in pre-clinical studies, some of them already in Phase 1. These companies are listed in Table 5.

A detailed description of some of the most important companies mentioned above is given below.

### 5.1. Aegle Therapeutics

Aegle Therapeutics is a biotechnological company from Miami, Florida, USA, and is the first for which human clinical testing for an exosome product has been approved by the U.S. Food and Drug Administration (FDA) for AGLE-102. Exosomes isolated from allogeneic BM-MSCs are used to treat dystrophic epidermolysis bullosa (DEB), i.e., a rare genetic pediatric connective tissue disorder, as well other serious dermatological disorders such as severe burns and wounds. Therapy can be performed by local injection or topically [193].

### 5.2. Capricor Therapeutics

Capricor Therapeutics is a clinical-stage biotechnology company from Beverly Hills, California, USA, and is one of the leading companies researching the field of exosomes. Researchers at Capricor Therapeutics are focused on developing and researching exosome-based therapeutics (Table 6) to treat and prevent severe and rare diseases and disorders, particularly Duchenne muscular dystrophy (DMD). They are also involved in the development of vaccines and the treatment of inherited diseases. One of them is already in Phase 1 development [197].

### 5.3. Codiak Biosciences

Codiak Biosciences from Cambridge, United Kingdom, has developed the engEx platform, which enables the production of exosomes with different properties, loaded with various therapeutic agents, and the ability to reach the desired target cells. They are developing various promising therapeutic exosomes based on engEx for the treatment of different types of cancer, neurological diseases and for vaccine development, as shown in Table 7. Two of these (exoIL-12™ and exoSTING™) are already in Phase 1 development, which began in September 2020 [200].

### 5.4. Evox Therapeutics

Evox Therapeutics is a company from Oxford, United Kingdom, that has developed the DeliverEX^TM^ platform, designed for the development of exosome-based therapeutics for the treatment of rare, life-threatening diseases. They appropriately modify exosomes, incorporated with drugs, to deliver these drugs to target organs to treat severe rare genetic disorders, including argininosuccinic aciduria (ASA), Citrullinemia type I, and phenylketonuria (PKU) [202]. They have already developed a few products, as shown in Table 8.

### 5.5. Exogenus Therapeutics

Exogenus Therapeutics is a Portuguese company and a pioneer in the development of exosome-based therapeutics. For their leading candidate, Exo-101, they have demonstrated in vitro and in vivo regenerative, anti-inflammatory, and immunomodulatory properties. Additional research is focused on the potential treatment of inflammatory skin diseases, such as psoriasis, and helping patients with inflammatory lung diseases, including COVID-19 patients with respiratory complications, and it has already been shown to accelerate the healing of chronic wounds. Exo-101 are isolated by an optimized process combining ultracentrifugation and size-exclusion pooling chromatography from umbilical cord blood mononuclear cells. They have also developed ExoWound, a combination of Exo-101 with a slow-release hydrogel that has potential for treating chronic wounds as it hardens at body temperature [210].

### 5.6. Anjarium Biosciences

Anjarium Biosciences is a company from Switzerland. They developed the Hybridosome^®^ platform for engineering exosome-based therapeutics production for the effective treatment and prevention of cancer and genetic diseases. In the development of engineering exosomes, they focus on both cargo incorporation and surface modifications to treat diseases for which current approaches are ineffective. Their focus is mainly on therapeutic RNA as the cargo. The product AB126 is both a therapeutic agent and a delivery vehicle to the central nervous system (CNS) to treat a variety of neurodegenerative diseases, such as Parkinson’s disease and multiple sclerosis, as well as for stroke, for which it is already in the preclinical phase [195].

### 5.7. Aruna Bio

Aruna Bio is a Greek company which in the past focused on the production of neural stem cells, but today mainly focuses on cell-free biological therapeutics, i.e., neuronal exosomes isolated from neuronal stem cells. They are developing a completely new platform—a neuronal exosome platform to deliver drugs as a new and effective way of treating neurodegenerative diseases. Their neuronal exosomes successfully target cells in the CNS, and in preclinical studies they have successfully achieved their crossing of the blood–brain barrier [196].

### 5.8. ReNeuron

ReNeuron is a company from the United Kingdom and is a leader in the field of clinical-stage stem cells. In addition to developing new stem cell therapies, they have also developed a new product, i.e., a therapeutic candidate, called ExoPr0, produced from CTX neural stem cells, which is a successful delivery vehicle as already proven in preclinical studies. It was developed for the treatment of neurodegenerative diseases, cancer, and for the development of vaccines such as COVID-19 [214].

### 5.9. Exopharm Pty Ltd.

Exopharm is an Australian clinical-stage company that develops exosome-based therapeutics. They are developing engineering exosomes for drug delivery in the treatment of infectious diseases, neurological diseases, and cancer, and natural exosomes, produced from stem cells and platelets, suitable for the treatment of osteoarthritis, chronic and acute injuries. Several products have already been developed (Table 9), some are in preclinical studies, and Plexaris^TM^ is already in Phase 1 [206].

### 5.10. ExoCoBio

ExoCoBio is a company from Seul, South Korea, and is one of the world’s leading companies in the production of exosome-based biomedical and regenerative therapeutics. They have patented technology for isolating and purifying exosomes from stem cells, technology for mass-production of highly efficient exosomes suitable for biomedical purposes, as well as optimized technology for regenerative medicine (ExoSCRT^TM^). Their focus is on the development of therapeutics for the treatment of atopic dermatitis, inflammatory bowel disease, acute kidney injury, and alopecia (hair loss), as well as for immuno-oncology treatment. They have also developed their line of exosome-based cosmetics Advanced Skincare Complex for Everyone (ASCE), and a cosmetic ingredient, hybrid exosome Vexosome^TM^ [205].

## 6. Conclusions and Future Perspectives

Exosomes have received a lot of attention over the last two decades, due to their unique characteristics, biocompatibility and safety, remaining stable through digestive and other biological fluids, and able to cross the blood–brain barrier. They are secreted by many different cells, and researchers have mainly focused on human, animal, and plant-derived exosomes. They are promising for use as biomarkers and in gene therapy, tissue regeneration, and vaccine development, and especially as delivery vehicles of drugs and other therapeutic agents. Extremely successful therapeutic effects of exosomes isolated from a variety of cells have been demonstrated in various studies in animal models.

By modifying naturally isolated exosomes, the most suitable nanocarriers can be prepared for the specific purpose of treatment and delivery of the desired therapeutic agents. The surface of exosomes can be modified to reach the recipient cells successfully and to enable an easy uptake into the cells. The desired components with therapeutic properties can be encapsulated into exosomes, which would not reach the target cells in such a high concentration without appropriate nanocarriers. However, it is also important to emphasize that these approaches must ensure that the structure and functions of isolated exosomes do not change significantly. Different methods can be used for exosome preparation. Knowing the advantages and disadvantages of individual methods of exosome production and preparation, which are also presented in detail in this review article, can greatly contribute to the selection of the most appropriate method for the synthesis of the desired exosomes. Further, new approaches have been developed for fully synthetic exosome production that mimics the characteristics of natural exosomes and could have more specific target properties, although these have not yet been explored to such an extent as modified exosomes. 

Current drugs and treatments for cancer can cause side effects, cytotoxicity, and long-term complications, and can also quickly lead to drug resistance in treated patients. As a result, exosomes are of great importance in the development of treatments for various types of cancer, as anti-tumor therapeutic efficacy has been successfully demonstrated in various in vitro and in vivo studies in animal models. They have also shown great potential as drug carriers for the possible treatment of various neurodegenerative and cardiovascular diseases and are effective in delivering antibiotics to pathogen-infected cells and thus have antibacterial activity.

Therapeutic biomaterials have recently played a particularly important role in the development of vaccines, especially for cell-free vaccines, which can be successfully achieved with the use of exosomes. A few global commercial companies (Aethlon Medical, Inc., Capricor Therapeutics, and ReNeuron), on which a detailed review was done for the first time in this work, are already developing vaccines that have potential for treating SARS-CoV-2 infections, but these are still only in the development phase.

Despite many successful studies, there are still many challenges to be overcome before exosome-based therapeutics can be used, as more clinical studies are needed. However, many companies have already developed appropriate exosome platforms and exosome products that are approaching clinical trial approvals or are already in Phase 1. Therefore, extremely successful drug delivery and effective treatment of various serious disease conditions can be achieved through the use of therapeutic biomaterials, i.e., exosomes, with these being highly promising approaches thar are likely to be increasingly important in the future.

## Figures and Tables

**Table 1:** The main advantages and disadvantages of the various methods for synthetic exosome production [9,14,16,18,20,85,92,97,98].

Methodology	Cell-Based Methodologies	Lipid Membrane Bilayer Formation Methodologies
Advantages	Suitable for mass production Production of exosomes similar to natural exosomes Immunotolerant, due to their origin from cells	Production of extremely pure nanoparticles with the desired composition (high pharmaceutical grade products) Suitable for the study of the individual elements
Disadvantages	Time-consuming Need for purification protocol Hard to control the production process	Use of extremely expensive lipids with high purity Risk of losing protein functions during production The need for deep knowledge of the composition of exosomes

**Table 2:** Examples of different types of cargo incorporated into naturally derived exosomes through modification methods.

Incorporated Cargo	Exosome Source	Modification Method	Results	Ref.
Hydrophobic Cargo (Incorporated Into Membrane)				
Curcumin	Murine macrophage RAW264.7 cells	Co-incubation	Good stability, inflammation-specific targeting ability, antioxidant features	[104]
Paclitaxel	U-87 cell	Co-incubation	Toxicity effect against glioblastoma multiforme cells	[105]
		Sonication		
	M1-macrophages	Sonication	Enhanced antitumor effects	[106]
Doxorubicin	HEK293 cell line	Electroporation	Rapid uptake into recipient cells, increased potency	[107]
Aspirin	Breast and colorectal cancer cells	Modified freeze–thawing process	Enhanced cellular uptake, improved cytotoxicity, anti-tumor effects	[108]
Hydrophilic Cargo (Incorporated Into Core)				
Melatonin	MSCs	Co-incubation (pre-isolation method)	Improved kidney recovery and function	[76]
Doxorubicin hydrochloride	BM-MSCs	Co-incubation	Cytotoxicity in osteosarcoma cells	[114]
Gemcitabine	Pancreatic cancer cells (Panc-1)	Co-incubation Sonication	Improved cellular uptake, therapeutic efficacy against pancreatic cancer, minimal damage to normal tissues	[109]
Linezolid	Mouse RAW 264.7 cells	Co-incubation	Efficacious intracellular antibiotic delivery	[110]
Vancomycin and lysostaphin	RAW264.7 cells	Sonication	Antimicrobial efficiency	[84]
Other Cargo				
Imperialine	Human plasma	Micelle-aided loading method	Increased antitumor effects	[115]
siRNA	Bovine milk	Electroporation Chemical transfection	Anti-tumor efficacy	[116]
	Breast cancer cell	Co-incubation	Suppression of postoperative metastasis (in triple negative breast cancer)	[117]
	Human normal MRC-5 fibroblasts cells Monkey normal Vero epithelial cells	Co-incubation	Increased cellular uptake efficiency	[118]
Hollow gold nanoparticles	Murine melanoma cells	Electroporation Diffusion Thermal shock Sonication Saponin-assisted loading	High encapsulation yield	[119]
c(RGDyK) peptide and curcumin	BM-MSCs from mice	Click chemistry	Suppression of the inflammatory response and cellular apoptosis in the lesion region	[90]

**Table 3:** Plant-based exosomes and their potential use as drug delivery vehicles.

Exosome Source	Incorporated Cargo	Results	Ref.
Strawberry	-	Strawberry-derived exosomes have been taken up by human MSCs. There was no trace of cytotoxicity, they even prevented oxidative stress in the human cells	[74]
Ginger	Doxorubicin	Effective inhibition of tumor growth in the Colon-26 xenograph tumor model	[120]
	Naturally occurring components: lipids, proteins, mRNA, 6-gingerol and 6-shogaol	Reduction in acute colitis, enhanced intestinal repair, and prevented chronic colitis and colitis-associated cancer. Potential for preventing inflammatory bowel disease	[121]
	-	Inhibition of NLRP3 inflammasome assembly and activation	[122]
Grapefruit	Inflammatory chemokine receptor, doxorubicin, or curcumin	Inhibition of tumor growth, inhibition of inflammatory effects of dextran sulfate sodium-induced mouse colitis	[123]
Broccoli	Sulforaphane	Prevention of DSS-induced colitis in B6 mice	[124]
Turnip	-	Inhibition of MCF-7 cells proliferation	[125]
Lemon			
Apple	Naturally occurring microRNA	Decreased OATP2B1 expression in human epithelial colorectal adenocarcinoma (Caco-2) cells at the 4levels of mRNA, protein content, and transport activity	[126]

**Table 4:** Drug delivery through different administration routes for various therapeutic purposes.

Source of Therapeutic Biomaterial	Incorporated Therapeutic Compound	Target	Administration Route	Disease/Condition	Therapeutic Effect	Ref.
BM-MSCs	-	Brain cells	Intranasal	Autism spectrum disorders (ASD)	Reduced symptoms of ASD	[179]
	Galectin-9 siRNA and oxaliplatin	PANC-02 cells	Intravenous	Pancreatic ductal adenocarcinoma (PDAC)	Antitumor efficacy	[83]
BM-MSCs from mice	c(RGDyK) peptide and curcumin	Lesion region of the ischemic brain—microglia, neurons, and astrocytes	Intravenous	Cerebral ischemia	Suppression of the inflammatory response and cellular apoptosis	[90]
Human adipose tissue-derived MSCs	-	Skin lesions	Intravenous Subcutaneous	Atopic dermatitis (AD)	Reduced pathological symptoms	[175]
Adipose-derived stem cells (ASC)	-	Glial cells	Intravenous Intranasal	Amyotrophic lateral sclerosis (ALS)	Improvement of motor performance; protective effect on lumbar motoneurons, neuromuscular junction and muscle, reduction in glial cells activation	[185]
Genetically modified dental pulp MSCs (DPSCs)	Tumor suppressor miR-34a	Breast carcinoma cells	In Vitro	Breast cancer	Anticancer effects	[151]
Bovine milk	siRNA	Lung cancer cells	Oral	Lung cancer	Antitumor efficacy	[116]
	hsa-miR148a-	Hepatic (HepG2) and intestinal (Caco-2) human cell lines	In Vitro	RNA-based therapy	Cost-effective source of exosome as nanocarriers	[186]
	Paclitaxel	Lung cancer cells	Oral	Lung cancer	Tumor growth inhibition	[177]
	Curcumin	Cervical cancer cells	Oral	Human cervical cancer	Antitumor activity	[187]
Bovine serum	α-d-mannose	Lymph nodes	Intradermal	Immunotherapy	Efficient delivery of immune stimulators and antigens	[188]
Human cardiac-resident mesenchymal progenitor cells (CPCs)	-	Cardiomyocytes	Intravenous	Cardiac toxicity	Inhibition of oxidative stress, prevention of myocardial fibrosis, inhibition of cell death	[189]
Human Wharton’s jelly MSC	Infrared-label	Corpus callosum, external capsule	Intranasal	Perinatal brain injury (PBI)	Neuroprotective effects	[180]
Macrophage	PLGA nanoparticles, loaded with doxorubicin	Tumor cells	Intravenous	Triple-negative breast cancer (TNBC)	Improvement of the cellular uptake efficiency and the antitumor efficacy, remarkable tumor-targeting efficacy, increased inhibition of tumor growth and induced intense tumor apoptosis	[165]
Mouse RAW264.7 macrophages	Linezolid	MRSA WHO-2-infected cells	Subcutaneous	MRSA infection	Efficacious intracellular antibiotic delivery	[110]
RAW264.7 macrophage cell line, mouse serums, human serums, and fetal bovine serums	CpG oligodeoxynucleotides (CpG ODN), ovalbumin (OVA), monophosphoryl lipid A (MPLA)	Macrophages in lymph nodes	Subcutaneous	Immune stimulation	Increased intracellular delivery, potential immune stimulation	[181]
RAW264.7 cells	Vancomycin and lysostaphin	MRSA WHO-2-infected cells	Intravenous	MRSA infection	Efficient antibiotic delivery, antibacterial efficiency	[84]
Mouse blood reticulocytes	Dopamine	bEnd.3 cells	Intravenous	Parkinson’s disease	Strong therapeutic efficacy, reduced systemic toxicity	[174]
HEK293T cell	Doxorubicin	PC3 prostate cancer cells	Intratumoral	Prostate cancer	Efficient tumor growth inhibition	[182]
Membrane protein from red blood cells and MCF-7 cancer cells	Doxorubicin	MCF-7 cells	Subcutaneous	Cancer	Anti-tumor therapeutic effect	[92]

**Table 5:** Commercial companies using exosomes.

Company Name	Commercial Exosome/Technology	Therapeutic Application	Exosome Source and Incorporated Cargo	Ref.
Aegle Therapeutics	AGLE-102	Serious dermatologic disorders	Allogeneic BM-MSCs	[193]
Aethlon Medical, Inc.	Hemopurifier^®^	Infectious disease and cancer	-	[194]
Anjarium Biosciences	Hybridosome^®^ platform	Cancer and genetic diseases	-	[195]
Aruna Bio	Neuronal exosome platform: product AB126	Neurological diseases	Neural stem cells	[196]
Capricor Therapeutics	See Table 6.	Severe and rare disorders (i.e., DMD)	-	[197]
Carmine Therapeutics	REGENT^®^	Gene therapy	Red blood cells	
Ciloa	Vaccine candidates against Chikungunya virus (FUI granted) and Zika	Antibodies, vaccines, therapeutic vectors	Recombinant exosomes	[198]
Clara Biotech	ExoRelease™	Exosome Isolation Platform	-	[199]
Codiak Biosciences	engEx™ platform (Products: see Table 7.)	Cancer, neurological diseases, vaccine development	-	[200]
Direct Biologics	ExoFlo^TM^	Providing signaling proteins that modulate inflammation	Human BM-MSCs	[201]
Evox Therapeutics	DeliverEX^TM^ platform (Products: see Table 8.)	Severe rare genetic disorders	Drug-loaded exosomes	[202]
EV Therapeutics Inc.	mTEV platform (EV101, EV102, EV103)	Gastrointestinal cancer, organ transplant rejection	-	[203]
Exocel Bio	EXOVEX	Regenerative medicine	-	[204]
ExoCoBio	ExoSCRT^TM^	Isolation and purification technology, technology for mass production of highly efficient exosomes	Stem cells	[205]
	Therapeutic and cosmetic products ASCE	Regenerate or activate/de-activate various tissues or cells		
Exopharm Pty Ltd.	Exopharm’s LEAP Technology	Isolating and purifying exosomes from adult stem cells	Stem cells	[206]
	Engineering exosomes (See Table 9.)	Antiviral, neurodegeneration, cancer	Cargos: such as RNA, enzymes and/or small molecules	
	Natural exosomes (See Table 9.)	Wound healing, osteoarthritis	Adult stem cells and platelets	
Exosome Diagnostics	ExoDx™ Prostate test	Diagnosis and assessment of the risk of prostate cancer	-	[207]
Exosome Sciences	TauSome™ biomarker	Diagnosis and monitoring of Alzheimer’s disease, chronic traumatic encephalopathy, and other neurological disorders	-	[208]
Exosomics Siena SpA	Exosome-based liquid biopsy	Exosome-based cancer screening and diagnosis	-	[209]
Exogenus Therapeutics	Exo-101	Regenerative medicine and inflammatory disorders (inflammatory skin conditions, inflammatory lung disorders, chronic wounds)	Umbilical cord blood mononuclear cells	[210]
Ilias Biologics Inc.	EXPLOR™ platform technology	Loading of specific proteins into exosomes in a controllable way	-	[211]
	Exo-Target^®^	Inflammatory and metabolic diseases, cancers	Therapeutic exosomes loaded with API molecule	
Kimera Labs	XoGlo^®^	Wound healing and skin rejuvenation/regeneration	MSCs	[212]
OmniSpirant	Inhaled exosome technology platform	Currently incurable respiratory diseases, cystic fibrosis	Bioengineered stem cells	[213]
Paracrine Therapeutics	Exosome Technology Platform	Regenerative medicine	Stem cells	
ReNeuron	ExoPr0	Neurodegenerative diseases, cancer, development of vaccines	CTX neural stem cells	[214]
Stem Cell Medicine Ltd.	Exosome-based technology	Neurodegenerative and neuropsychiatric indications: autism spectrum disorder (ASD)	Adult stem cells	[215]
TAVEC Pharmaceuticals	miRNA-loaded exosomes	Anti-cancer gene therapy	-	[216]
XOStem Inc.	XO-Regen^®^	Articular damage, respiratory failure, neuroinflammation	Bone marrow and umbilical cord derived MSCs	[217]
	XO-Cutis^®^	Hair regeneration, skin rejuvenation, wound healing		

**Table 6:** Promising therapeutic candidates from Capricor Therapeutics [197].

Therapeutic Candidate	Purpose	Development Phase
Exosome mRNA Vaccine (Tripartite mRNA design)	SARS-CoV-2	Preclinical
Exosome VLP Display Vaccine (4-part antigen design)	SARS-CoV-2	Preclinical
CDC-Exosomes (allogenic cardiosphere-derived cells (CDC)-exosomes)	DMD	Phase 1
Engineered Exosomes (RNA delivery)	Evaluating	Discovery
ASTEX-Exosomes (engineered fibroblast-derived exosomes)	Evaluating	Discovery

**Table 7:** Exploring engEx therapeutic candidates from Codiak Biosciences [200].

Field	Therapeutic Candidate	Purpose	Administration Route	Development Phase
Oncology	exoIL-12™	Cutaneous T-cell lymphoma (CTCL), Melanoma, Triple-negative breast cancer (TNBC), Merkel cell carcinoma (MCC), Kaposi’s sarcoma, Glioblastoma multiforme (GBM)	Intratumoral	Phase 1
	exoSTING™	Solid tumors, i.e.,: Head and neck squamous cell Carcinoma (HNSCC), TNBC, Anaplastic thyroid cancer (ATC), Cutaneous squamous cell carcinoma (cSCC)	Intratumoral	Phase 1
	exoSTING™	GMB, Leptomeningeal cancer (LMD)	Intratumoral, intratheca	Preclinical
	exoASO™-STAT6	Myeloid rich cancers, i.e.,: Hepatocellular carcinoma (HCC), Pancreatic ductal adenocarcinoma (PDAC), Colorectal cancer (CRC) Ovarian cancer	Intratumoral	Preclinical
	exoASO™-STAT3	Hematologic/hepatic cancers	TBD	Preclinical
	exoASO™-NRAS	Hematologic cancers/solid tumors	TBD	Discovery
	Oncogene Targets	Hematologic cancers/solid tumors	TBD	Discovery
Neurology	exoASO-NLRP3	Neuroinflammation	Intrathecal	Discovery
	exoASO™-NLRP3	Neuropathy	Intrathecal	Discovery
	Gene Targets	Neuromuscular diseases	TBD	Discovery
Vaccines	exoVACC™	Cancers, neurodegenerative diseases, viral diseases	TBD	Discovery

**Table 8:** Exosome-based therapeutics from Evox Therapeutics [202].

Field	Therapeutic Product	Purpose	Cargo	Development Phase
Urea cycle disorders	EVX-102	ASA	Protein exosomes	Pre-clinical
	EVX-103	Citrullinemia type I	Protein exosomes	Discovery
Rare metabolic	-	PKU	Undisclosed modality	Discovery

**Table 9:** Therapeutic products from Exopharm Pty Ltd. [206].

Exosome Type	Therapeutic Product	Purpose	Development Phase
Natural exosomes	Plexaris	Wound healing	Phase 1
	Cevaris	Osteoarthritis	Pre-clinical
Engineering exosomes	Fortrexo	Antiviral	Pre-clinical
	Cognevo	Neurodegeneration	Discovery
	PlexoDOX	Cancer	Discovery
